# Supplementary material for: Feature harmonization reveals that apparent cross-cohort differences in tau-positivity prediction reflect variable availability and cohort stage structure
Source: medRxiv. 2026 Oct 21:2026.10.16.26365770. Preprint. [Version 1] doi: 10.64898/2026.10.16.26365770 (PMC13738908; doi:10.64898/2026.10.16.26365770)
Supplement: Supplementary file 1 [file NIHPP2026.10.16.26365770V1-supplement-1.pdf]

medRxiv preprint doi: https://doi.org/10.64898/2026.10.16.26365770; this version posted October 21, 2026. The copyright holder for this preprint (which was not certified by peer review) is the author/funder, who has granted medRxiv a license to display the preprint in perpetuity.
It is made available under a CC-BY 4.0 International license .

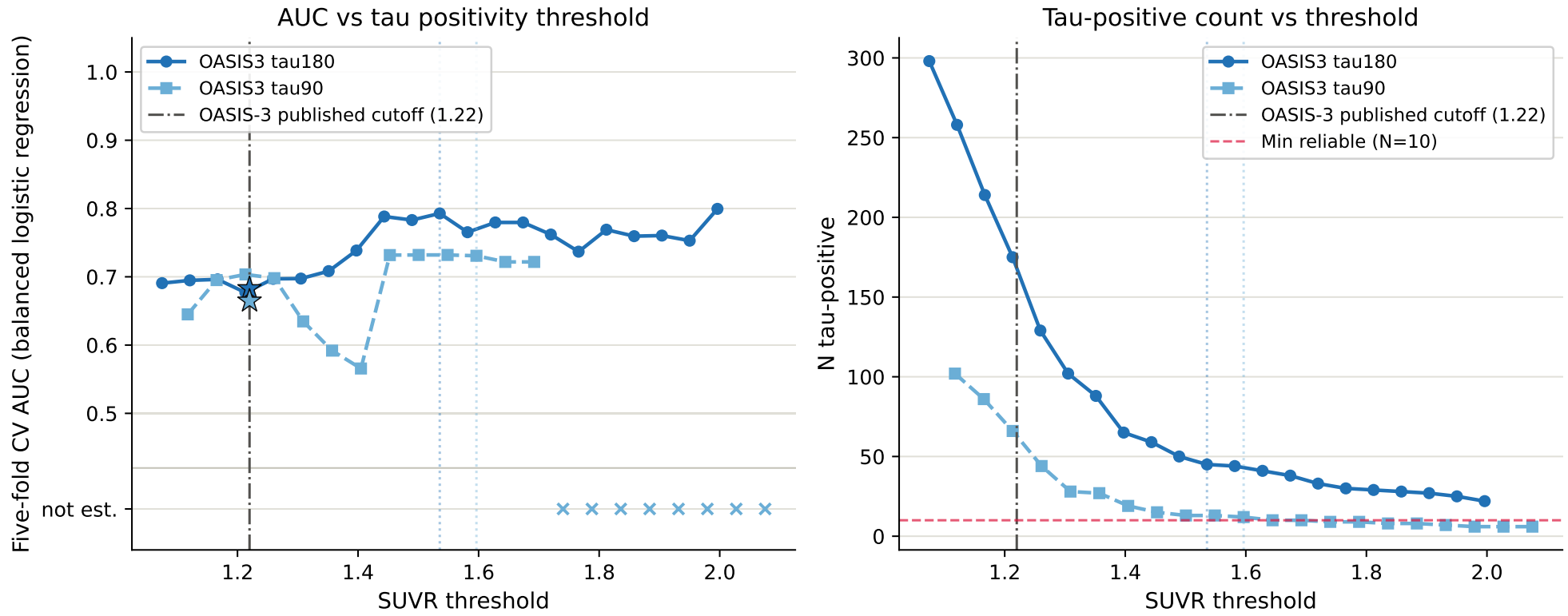


**Fig. S1 OASIS3 tau-threshold sensitivity analysis.** Threshold-sensitivity analysis for OASIS3 tau positivity using demographic/genetic predictors only: age, APOE $\varepsilon4$ count, APOE $\varepsilon4$ carrier status, education, and sex. Left, AUC versus threshold; right, number of tau-positive subjects versus threshold. Vertical dotted lines mark the cohort-specific Gaussian mixture model midpoint thresholds used to derive tau positivity; the dash-dotted line and stars mark the published OASIS-3 cutoff (1.22). Points indicate mean five-fold cross-validated AUC from balanced logistic regression at each threshold. Thresholds yielding fewer than 10 tau-positive subjects (red dashed line, right) are marked × in the 'not est.' band. In OASIS3 tau180, demographic discriminability remained directionally stable near the GMM midpoint, where the tau-positive count matched the primary cohort definition ($n$ = 45). In OASIS3 tau90, the GMM midpoint yielded 12 tau-positive subjects and a demographic/genetic AUC of 0.731, but the cohort remained too small for primary inference.

**Table S5** Selected OASIS3 threshold-sweep results around the GMM midpoint using the harmonized tauopathy SUVR variable. AUCs are from demographic/genetic-only balanced logistic regression using age, APOE $\varepsilon4$ count, APOE $\varepsilon4$ carrier status, education, and sex. The first row of each block uses the published OASIS-3 tauopathy cutoff (1.22) [16]. Reliable thresholds are defined as those with at least 10 tau-positive subjects. AUCs use a single five-fold split (seed 42), as in the primary analysis; across 100 split seeds the standard deviation at the thresholds checked was about 0.01 (tau180) and 0.02 (tau90).

| Cohort | % from GMM | Threshold | $N$ | Tau+ | AUC | Reliable |
|---|---|---|---|---|---|---|
| OASIS3 tau180 | −20.6% (published) | 1.220 | 368 | 171 | 0.683 | Yes |
| OASIS3 tau180 | −9% | 1.397 | 368 | 65 | 0.738 | Yes |
| OASIS3 tau180 | −6% | 1.443 | 368 | 59 | 0.788 | Yes |
| OASIS3 tau180 | −3% | 1.489 | 368 | 50 | 0.783 | Yes |
| OASIS3 tau180 | 0% | 1.536 | 368 | 45 | 0.793 | Yes |
| OASIS3 tau180 | +3% | 1.582 | 368 | 44 | 0.765 | Yes |
| OASIS3 tau180 | +6% | 1.628 | 368 | 41 | 0.780 | Yes |
| OASIS3 tau90 | −23.6% (published) | 1.220 | 142 | 64 | 0.665 | Yes |
| OASIS3 tau90 | −9% | 1.453 | 142 | 15 | 0.732 | Yes |
| OASIS3 tau90 | −6% | 1.501 | 142 | 13 | 0.732 | Yes |
| OASIS3 tau90 | −3% | 1.549 | 142 | 13 | 0.732 | Yes |
| OASIS3 tau90 | 0% | 1.596 | 142 | 12 | 0.731 | Yes |
| OASIS3 tau90 | +3% | 1.644 | 142 | 10 | 0.722 | Yes |
| OASIS3 tau90 | +6% | 1.692 | 142 | 10 | 0.722 | Yes |
| OASIS3 tau90 | +9% | 1.740 | 142 | 9 | — | No |

medRxiv preprint doi: https://doi.org/10.64898/2026.10.16.26365770; this version posted October 21, 2026. The copyright holder for this preprint (which was not certified by peer review) is the author/funder, who has granted medRxiv a license to display the preprint in perpetuity.
It is made available under a CC-BY 4.0 International license .

**Table S6** Standardized logistic regression coefficients from the primary matched-feature reference-combined model, fitted on the full cohort. Numeric features, including the binary APOE $\varepsilon4$ carrier indicator, were median-imputed and z-scored, so coefficients are changes in log-odds per one-SD increase; sex is the difference between the female and male one-hot coefficients (log-odds, not per SD). CDR-SB had the largest coefficient in every cohort (1.37–1.69). In ADNI it was more than twice as large as any demographic/genetic coefficient (largest: APOE $\varepsilon4$ count, 0.49–0.59); in OASIS3, age (1.33) and APOE $\varepsilon4$ count (1.07) approached CDR-SB, consistent with the earlier disease-stage composition of that sample. APOE $\varepsilon4$ carrier status and allele count are highly collinear ($r = 0.92$–$0.93$), so their individual coefficients (including the negative OASIS3 carrier coefficient) should not be interpreted separately. The negative FAQ coefficient after conditioning on CDR-SB likewise reflects collinearity between the two severity measures.

| Feature | ADNI tau90 | ADNI tau180 | OASIS3 tau180 |
|---|---|---|---|
| CDR-SB | 1.37 | 1.40 | 1.69 |
| Age | 0.23 | 0.15 | 1.33 |
| APOE $\varepsilon4$ carrier | 0.25 | 0.15 | −0.16 |
| APOE $\varepsilon4$ count | 0.49 | 0.59 | 1.07 |
| Education | −0.18 | −0.15 | 0.08 |
| Sex (female vs. male)[a] | −0.18 | −0.15 | 1.02 |
| FAQ | −0.24 | −0.19 | −0.19 |

[a]Difference between the female and male one-hot coefficients (log-odds, not per SD).

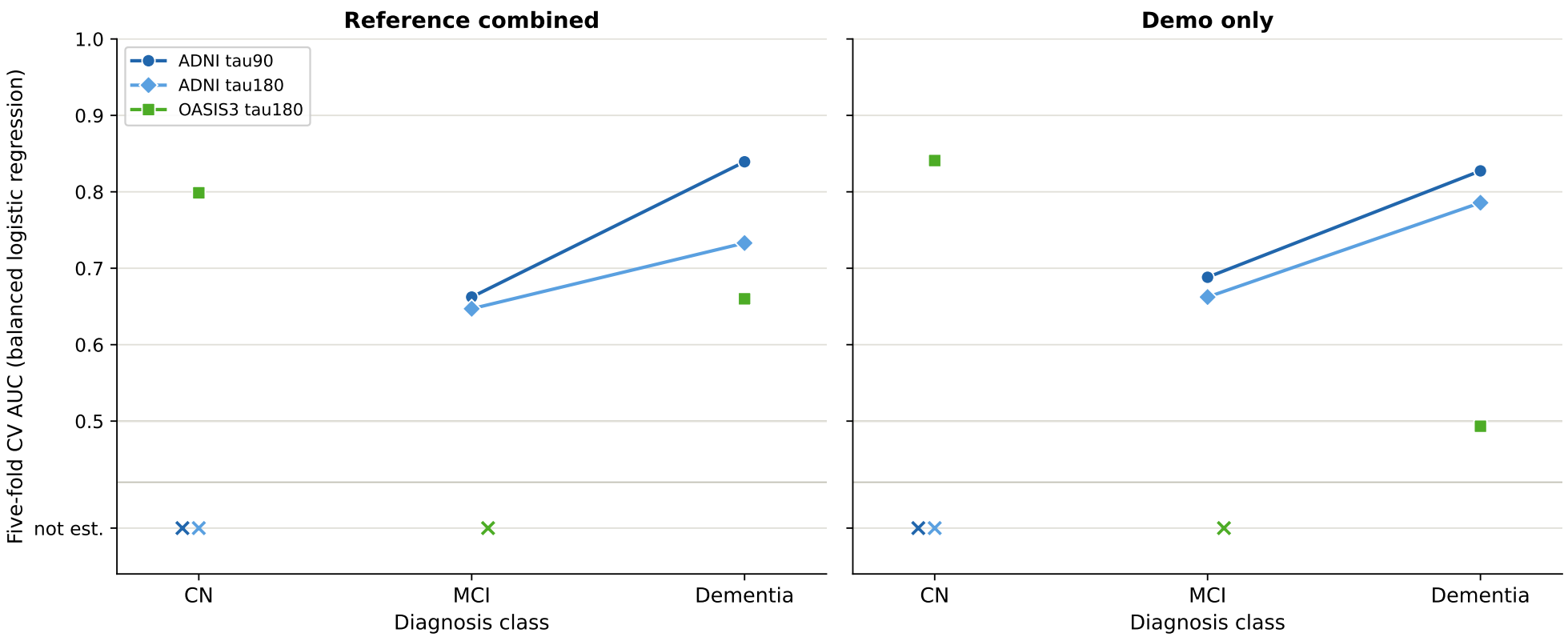


**Fig. S2 Diagnosis-stratified feature-block AUC analysis.** Reference-combined and demo-only AUCs stratified by baseline diagnosis (CN, MCI, Dementia) for ADNI tau90, ADNI tau180, and OASIS3 tau180 (balanced logistic regression, five-fold cross-validation). Strata with fewer than 10 tau-positive or 10 tau-negative subjects were not estimated and are marked × in the 'not est.' band (ADNI CN: $N_+ = 6$; OASIS3 MCI: $N_+ = 4$). In OASIS3 tau180, the CN stratum shows demo-only AUC (0.841) exceeding reference AUC (0.799), suggesting that severity features add noise when CDR-SB is near-uniformly zero; the Dementia stratum shows the pattern reversing (reference 0.660 vs. demo-only 0.493; $N_+ = 25$, $N_- = 12$), consistent with severity contributing discriminative information when functional impairment is present. In ADNI, the severity advantage in the Dementia stratum was present in tau90 (reference 0.839 vs. demo-only 0.827) but directionally inconsistent in tau180 (reference 0.733 vs. demo-only 0.786), indicating a weaker and less consistent effect than in OASIS3.

medRxiv preprint doi: https://doi.org/10.64898/2026.10.16.26365770; this version posted October 21, 2026. The copyright holder for this preprint (which was not certified by peer review) is the author/funder, who has granted medRxiv a license to display the preprint in perpetuity.
It is made available under a CC-BY 4.0 International license .

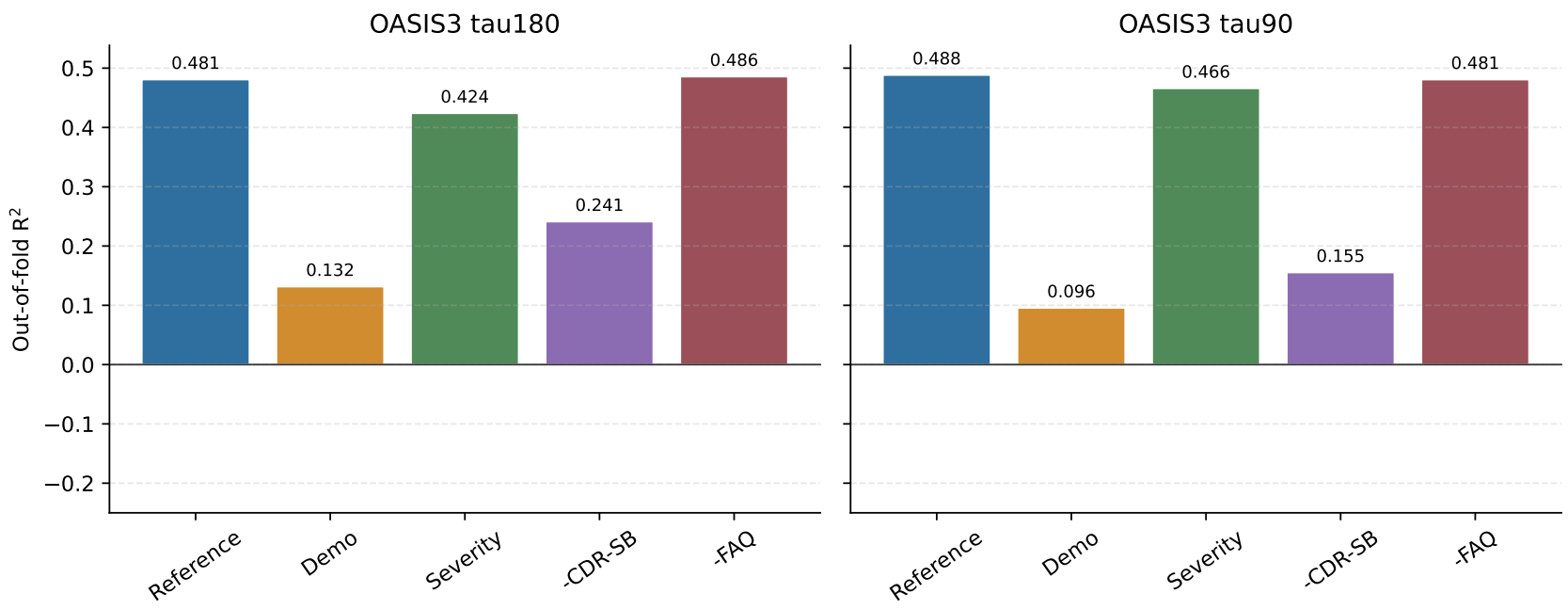


**Fig. S3 Within-OASIS3 continuous tauopathy SUVR sensitivity analysis.** Cross-validated $R^2$ for ridge-regression models predicting continuous Tauopathy SUVR from matched feature blocks in OASIS3 tau90 and tau180. The reference-combined and severity-only models explained substantially more variance than the demo-only model, indicating that the OASIS3 severity-stage signal persisted when tauopathy was modeled as a continuous outcome rather than binarized at the GMM midpoint.

**Table S7** Within-OASIS3 continuous tauopathy SUVR sensitivity analysis. Ridge-regression models were fit using continuous Tauopathy SUVR as the outcome and the same matched feature blocks as in the binary analysis. Performance is reported as five-fold cross-validated $R^2$. This analysis was performed only within OASIS3 and was not used for direct cross-cohort comparison.

| **Cohort** | **Reference** | **Demo-only** | **Severity-only** | **Minus all CDR*** | **Minus FAQ** |
|---|---|---|---|---|---|
| OASIS3 tau180 | 0.481 | 0.132 | 0.424 | 0.241 | 0.486 |
| OASIS3 tau90 | 0.488 | 0.096 | 0.466 | 0.155 | 0.481 |

*In the matched OASIS3 feature set, minus-all-CDR is operationalized as removing CDR-SB.
